# COMPARISON OF DYNESYS AND HYBRID SYSTEM FOR MULTI-SEGMENTAL LDD

**DOI:** 10.1590/1413-785220243202e270051

**Published:** 2024-06-24

**Authors:** Xin Zhang, Xiao Xiao, Hongyu Wang, Song Wang, Dazhi Yang, Songlin Peng

**Affiliations:** 1 The Second Clinical Medical College, Jinan University, Shenzhen, Guangdong, China.; 2 Shenzhen People's Hospital, Department of Orthopaedic Surgery, Shenzhen Division of Spine Surgery, Shenzhen, Guangdong, China.; 3 Shenzhen Key Laboratory of Musculoskeletal Tissue Reconstruction and Function Restoration, Shenzhen, Guangdong, China.; 4 Shenzhen People's Hospital, Shenzhen Clinical Research Centre for Geriatrics, Shenzhen, Guangdong, China.

**Keywords:** Intervertebral Disc Degeneration, Surgical Procedures, Operative, Comparative Study, Degeneração do Disco Intervertebral, Procedimentos Cirúrgicos Operatórios, Estudo Comparativo

## Abstract

**Objective:**

To compare effectiveness of Dynesys and hybrid system in treating patients with multi-segmental lumbar degenerative disease (LDD).

**Methods:**

Patients involved in this retrospective study were divided into Dynesys (n = 22) and Hybrid (n = 13) groups. Clinical outcomes were evaluated using Oswestry Disability Index (ODI), and Visual Analogue Scale (VAS). Radiologic evaluations included X-ray, MRI, and CT. Furthermore, different complications were analyzed.

**Results:**

At the last follow-up, ODI and VAS of each group were improved (p < 0.05), and the range of motion (ROM) of operating segments decreased. However, Dynesys group preserved a larger extent of ROM at the final follow-up (p < 0.05). ROM of the upper adjacent segment was increased in both groups (p < 0.05), while the disc heights were decreased at the final follow-up (p < 0.05). Besides, Dynesys group had a more obvious decrease in the disc height of dynamic segments (p < 0.05). No significant difference existed in complications between both groups (p > 0. 05).

**Conclusion:**

In our study, similar satisfactory results were obtained in both groups. Both surgical procedures can be employed as effective treatments for middle-aged and physically active patients with multi-segmental LDD. ***Level of Evidence III; Retrospective Comparative Study.***

## RESUMO

**Objetivo:**

Comparar a eficácia do Dynesys e do sistema híbrido no tratamento de pacientes com doença degenerativa lombar multissegmentar (DLD).

**Métodos:**

Os pacientes envolvidos neste estudo retrospectivo foram divididos em grupos Dynesys (n = 22) e Híbrido (n = 13). Os desfechos clínicos foram avaliados por meio do Oswestry Disability Index (ODI) e da Escala Visual Analógica (EVA). As avaliações radiológicas incluíram radiografia, ressonância nuclear magnética (RNM) e tomografia computadorizada. Ademais, diferentes complicações foram analisadas.

**Resultados:**

No acompanhamento final, o ODI e a EVA de todos os grupos melhoraram (p < 0,05), e houve diminuição da amplitude de movimento (ADM) dos segmentos operacionais. No entanto, o grupo Dynesys preservou uma maior extensão da ADM no acompanhamento final (p < 0,05). A ADM do segmento superior adjacente foi ampliada em ambos os grupos (p < 0,05), enquanto as alturas dos discos foram reduzidas no acompanhamento final (p < 0,05). No entanto, o grupo Dynesys apresentou uma redução mais evidente na altura do disco dos segmentos dinâmicos (p < 0,05). Não houve diferença significativa nas complicações entre esses dois grupos (p > 0,05).

**Conclusão:**

Neste estudo, resultados satisfatórios semelhantes foram obtidos em ambos os grupos. Ambos os procedimentos cirúrgicos podem ser empregados como tratamentos eficazes para pacientes de meia-idade e fisicamente ativos com LDD multissegmentar. ***Nível de Evidência III; Estudo Retrospectivo Comparativo.***

## INTRODUCTION

Of all the spinal diseases, lumbar degenerative disease (LDD) is the most common disease. It often develops into multi-segmental LDD over time. This disease generally responds well to conservative treatments, but some patients may need surgery due to severe back and leg pains. Spinal fusion are considered as the best surgical option for LDD, but most fixation devices are presently made of titanium alloy, which could cause many issues such as surgical site infection (SSI) and adjacent segment degeneration (ASD).^1-3^ Additionally, as the number of fused segments increases, so does the likelihood of ASD.^4^ Aside from that, physically active patients would have to give up their favorite sport after fusion surgery for limited lumbar spine mobility.

In light of these issues, researchers designed Dynesys to replace rigid fusion for treating LDD. It could preserve the mobility of the operated segment and lessen the pressure on the adjacent discs and facet joints.^5^ And studies have supported the beneft of Dynesys in preserving range of motion (ROM) and preventing ASD in LDD patients,^6^ which means that it can be installed in middle-aged patients with single- or multi-segmental LDD. However, other study argued that Dynesys failed to achieve that beneficial effect.^7^

When the patient suffers from multi-segmental LDD yet wishes to retain some spinal mobility for sports and other recreational activities, the surgeon has to carefully consider the surgical protocol. Hybrid fixation have been utilized in LDD patients with at least two affected segments because the degree of degeneration of each segment varies. Currently, there are two types of hybrid fixation systems in clinical practice. The Dynesys-Transition-Optima system with Dynesys Screw, Transition Screw, and Optima Screw, effectively treats multi-level LDD. Yet, its internal structure may lead to operational failure.^8^ In hybrid fixation, the dynamic segment is only fixed by the Dynesys device, whereas the fusion segment is fixed by both the Dynesys device and an intervertebral cage. Our team demonstrated in a previous study that the hybrid fixation device has comparable efficacy as rigid fusion in treating multi-segmental LDD within one year. However, hybrid fixation preserves spinal mobility better than rigid fusion.^9^

Researchers have primarily compared hybrid fixation to rigid fusion or Dynesys fixation to rigid fusion, neglecting a comprehensive comparison between Dynesys fixation and hybrid fixation. This study analyzed LDD patients undergoing multi-segmental hybrid fixation, contrasting them with a control group receiving Dynesys fixation. Our retrospective analysis aimed to assess clinical and radiological outcomes and complications of both techniques, shedding light on the most effective surgical approach for physically active middle-aged LDD patients.

## Patients and Methods

### Patient selection

The studies involving human participants were reviewed and approved by the Scientific Research Ethics Committee of Shenzhen People's Hospital (KY-LL-2021586-02). Informed consent was obtained from all subjects and/or their legal guardians.

Patient data were collected from January 2015 to August 2019. The inclusion criteria were: (1) diagnosed with lumbar disc herniation or lumbar spinal stenosis or both, via imaging; (2) had two or more affected segments; (3) symptoms persisted after six months of conservative treatment; (4) received Dynesys or Hybrid fixation. The exclusion criteria were: (1) severe osteoporosis (bone mineral density T-score < -2.5) in the lumbar spine; (2) severe spinal deformities such as Meyerding Grade II or higher spondylolisthesis, Cobb angle > 30°, and spinal rotation; (3) vertebral fracture, infection, tumor, and ankylosing spondylitis; (4) systemic connective tissue disease; (5) less than one year of recorded follow-up or incomplete follow-up records. A total of 35 patients with multi-segmental LDD were included.

### Operating technique

#### Dynesys fixation

After disinfection and draping, a midline incision was made on the back. Bilateral muscles were dissected along the supraspinous ligament. Dynesys pedicle screws (Zimmer, Switzerland) were implanted at the intersection of the lateral facet of the articular process and the root of the transverse process. Following laminectomy and removal of the ligamentum flavum, discectomy relieved impinged nerve roots. The cord was inserted through the spacer and the second pedicle screw sequentially. The LIS Cord Tensioner Set was placed over the Guide Wire atop the screw head. The cord was threaded through the LIS Cord Tensioner, snapping the spacer. Finally, the surgical site was irrigated and closed by layers.

#### Hybrid fixation

A longitudinal incision was made bilaterally along the supraspinous ligament, separating the muscle groups. Dynesys pedicle screws (Zimmer, Switzerland) were implanted on both sides of the operative segments. Laminectomy and discectomy were performed on non-fusion segments to decompress the spinal canal and nerve roots. For fusion segments, inferior and superior facet joints were removed. After further decompression, foraminotomy, and discectomy, cartilage endplates and discs were removed for ideal bone-to-bone surface. Bone tissues were inserted into appropriately sized cages (Johnson & Johnson, USA), then into intervertebral space. Connector and spacer installation followed the Dynesys group procedure. Finally, the surgical site was irrigated and closed by layers.

#### Clinical and radiographic evaluations

The following perioperative data were collected: operating duration, blood loss, drain volume, length of hospital stay, and postoperative length. The Oswestry Disability Index (ODI) and the Visual Analogue Scale (VAS) were assessed for clinical outcomes.

The patient's disc height (DH) was measured from standing lumbar spine X-ray images before surgery, one week after surgery, and at the final follow-up. The anterior intervertebral space height (AH), the central intervertebral space height (CH), and the posterior intervertebral space height (PH) were measured at the affected and upper adjacent segments. The DH was calculated: DH=(AH+CH+PH)/3 .

Before surgery and at the last follow-up, lumbar spine X-ray images were taken to determine the range of motion (ROM) of the operative and upper adjacent segments. ROM was defined as the amount of change in the Cobb angle in the flexion and extension views.

The lumbar spine MRI was taken prior to surgery and at the last follow-up, showed the Pfirrman grade of the operative and upper adjacent segments. The rate of intervertebral disc degeneration was evaluated using the following formula: the number of patients who had Pfirrmann grade degeneration after surgery/the number of the total patients× 100%.^9^

#### Surgical complications

The criteria described by Liu were used to diagnose SSI during the follow-up.^10^

In standing lumbar spine X-ray images and CT scans, screw loosening appears as a "double halo sign", described as a radiolucent rim surrounding the screw encircled by dense bone trabeculae. ASD is defined either radiographically or symptomatically as Zhang and Xiao's studies described.^6,9^

### Statistical Analysis

Statistical analysis was performed using SPSS version 26.0 (IBM, USA). The data were tested for normal distribution using the Kolmogorov-Smirnov test. Mann-Whitney U test, Wilcoxon signed-rank test, Kruskal-Wallis H test, and Friedman M test were used for continuous variables, while the Chi-square test was applied for categorical variables. *P* < 0. 05 was considered a statistically significant difference.

## RESULTS

A total of 35 patients with multi-level LDD were enrolled in this retrospective study, of which 22 received Dynesys fixation, and 13 received hybrid fixation. There was no significant difference in age, gender, BMI, follow-up time, operating levels, disease types, preoperative VAS, and preoperative ODI between the two groups (*p* > 0.05, Table 1).

**Table 1:** Demographic Characteristics.

	Dynesys group (n=22)	Hybrid group (n=13)	p
Age (years)	48.0 ± 10.0	56.5 ± 15.4	0.053
Gender (male/female)	15/7	9/4	1
BMI (kg/m^2^)	23.8 ± 2.8	24.7 ± 3.9	0.448
Follow-up time (months)	21.0 ± 7.3	18.0 ± 8.7	0.113
Operating levels (n)			0.541
Two levels	21	11	
More than two levels	1	2	
Diseases (n)			0.851
Spinal stenosis	1	1	
Lumbar disc herniation	5	4	
Spinal stenosis combined with lumbar disc herniation	16	8	

### Clinical outcomes

#### Perioperative data

There was no significant difference between the two groups concerning the length of hospital stay, Post-operation length of hospital stay, and drainage volume. However, the Hybrid group lost significantly more blood than the Dynesys group and had significantly longer surgical operations (*p* < 0.05, Table 2).

**Table 2:** Perioperative Data

	Dynesys group (n=22)	Hybrid group (n=13)	^p^
Operating duration (min)	192.6 ± 60.0	236.0 ± 55.3	0.012
Blood loss (mL)	174.0 ± 52.6	373.1 ± 164.1	0.001
Drainage volume (mL)	274.5 ± 248.1	357.7 ± 190.7	0.067
Length of hospital stay (days)	17.0 ± 8.1	18.0 ± 5.1	0.229
Post-operation length of hospital stay (days)	12.1 ± 7.1	12.3 ± 3.0	0.448

#### ODI and VAS

The ODI and VAS of both groups were significantly improved at the final follow-up than pre-operation (*p* < 0.05). There was no significant difference in ODI between the two groups at each time point (*p* > 0.05). However, the difference in VAS at the final follow-up between the two groups was statistically significant (*p* < 0.05, Table 3).

**Table 3:** ODI and VAS.

	Dynesys group	Hybrid group	^p^
**ODI (%)**			
Pre-operation	62.5 ± 10.5	62.9 ± 10.7	0.933
Final follow-up	23.5 ± 15.0*	18.1 ± 2.8*	0.775
**VAS**			
Pre-operation	6.8 ± 0.8	7.2 ± 0.9	0.257
Final follow-up	2.0 ± 2.2*	0.7 ± 0.9*	0.015

* Significant difference between pre-operation and final follow-up in each group, *p* < 0.05.

### Radiologic outcomes

#### ROM of affected segments and the upper adjacent segment

In both groups, the ROM of affected segments decreased at the last follow-up (*p* < 0.05). However, it was significantly higher in the Dynesys group than in the Hybrid group at the last follow-up (*p* < 0.05). The ROM of the upper adjacent segment increased in both groups (*p* < 0.05). There was no significant difference in the ROM of the upper adjacent segment between the two groups at each time point (*p* > 0.05, Table 4).

**Table 4:** ROM of operating segments and the upper adjacent segment.

	Dynesys group	Hybrid group	^p^
**ROM of operating segments (**°)			
Pre-operation	9.2 ± 6.1	11.5 ± 9.6	0.428
Final follow-up	6.4 ± 3.4*	4.4 ± 1.7*	0.029
**ROM of the upper adjacent segment (**°)			
Pre-operation	3.7 ± 2.1	3.3 ± 1.9	0.257
Final follow-up	6.4 ± 3.5*	5.4 ± 2.8*	0.169

* Significant difference between pre-operation and final follow-up in each group, *p* < 0.05.

#### DH of operating segments and the upper adjacent segment

In the Dynesys group, the DH of the operating segments dropped at the final follow-up (*p* < 0.05). In the Hybrid group, the DH of the dynamic segment increased one week after surgery (*p* < 0.05), then decreased at the final follow-up (*p* < 0.05). Also, in the Hybrid group, the DH of the fusion segment did not change significantly from pre-operation to one-week post-operation (*p* > 0.05) but did drop at the final follow-up (*p* < 0.05).

The DH of the upper adjacent segment in both groups one-week post-operation was significantly improved than pre-operation (*p* < 0.05). The DH in both groups significantly declined at the final follow-up than one-week post-operation (*p* < 0.05, Table 5).

**Table 5:** DH of operating segments and the upper adjacent segment.

	Dynesys group	Hybrid group		^p^
		Dynamic segment	Fusion segment	
**Disc height of stabilized segment (mm)**				
Pre-operation	11.2 ± 2.0	9.5 ± 1.4	9.9 ± 2.4	0.006
one week after surgery	12.1 ± 3.4	10.2 ± 1.9†	11.5 ± 2.1	0.064
Final follow-up	9.9 ± 1.9* #	8.7 ± 1.8*	9.7 ± 1.4*	0.175
**Disc height of the upper adjacent segment (mm)**				
Pre-operation	11.7 ± 1.9	9.8 ± 1.2		0.001
one week after surgery	12.6 ± 1.1†	10.6 ± 1.9†		0.002
Final follow-up	11.0 ± 0.9* #	9.4 ± 1.3*		0

† Significant difference between pre-operation and one-week post-operation in each group, *p* < 0.05.

* Significant difference between one-week post-operation and final follow-up in each group, *p* < 0.05.

# Significant difference between pre-operation and final follow-up in each group, *p* < 0.05.

#### Pfirrmann grade

At the final follow-up, the Dynesys group reported an average disc degeneration rate of 9.09%, while the Hybrid group reported 15.38%, without a significant difference between them (*p* > 0.05, Tables 6 and 7).

**Table 6:** Pfirrmann grade.

Preoperative	Final follow-up							
	Dynesys group (n)				Hybrid group (n)			
	II	III	IV	V	II	III	IV	V
II	–	–	–	–	–	–	–	–
III	–	15	2	–	–	7	2	–
IV	–	–	5	–	–	–	3	–
V	–	–	–	–	–	–	–	1

**Table 7:** Disc degeneration rate.

	Dynesys group (n)	Hybrid group (n)	Total
No degeneration	20	11	31
Degeneration	2	2	4
Total	22	13	35

P =0.618

#### Complications

One-week post-operation, SSI occurred in two patients in the Dynesys group. In the Hybrid group, only one patient experienced dysuria six days after surgery. It was believed that the patient had developed a urinary tract infection. Until the last follow-up, there was one case of screw loosening in the Dynesys group, but none in the Hybrid group. There were no symptomatic ASD cases. There were 5 cases of radiographic ASD in the Dynesys group and 3 cases in the Hybrid group (Table 8).

**Table 8:** Complication.

	SSI (n)	Screw loosening (n)	Radiologic ASD (n)	Symptomatic ASD (n)
Dynesys group	2	1	5	0
Hybrid group	0*	0*	3*	0*

* No significant difference between both groups, *p* > 0.05.

#### Typical Cases

**Figure 1:**
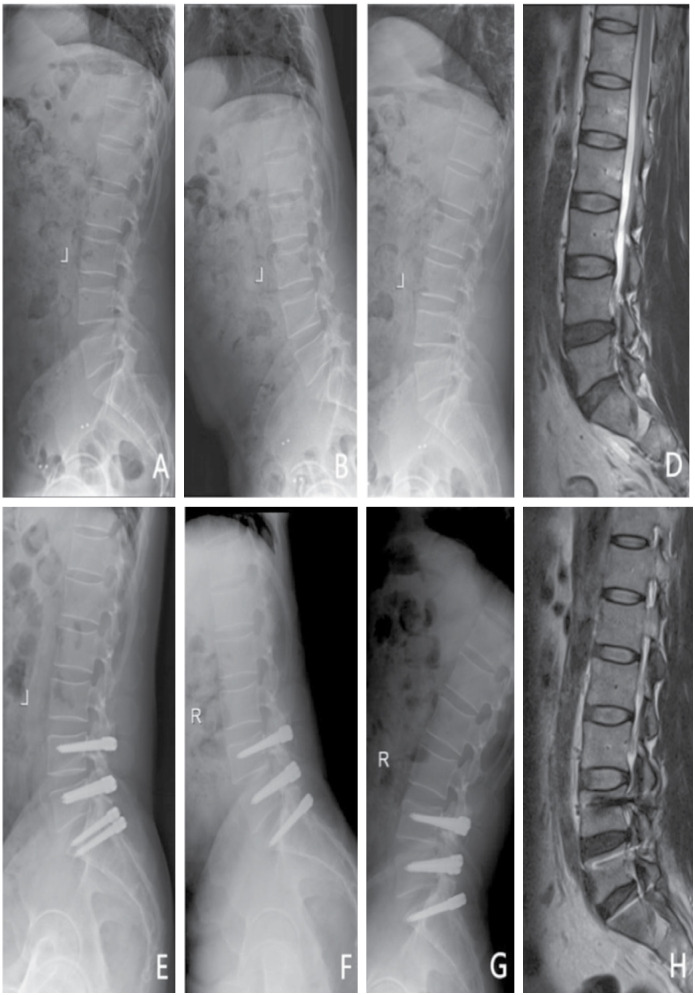
A 47-year-old male patient underwent surgery with Dynesys system due to lumbar disc herniation and spinal stenosis in L4/5 and L5/S1. (A) Pre-operation lateral X-ray. (B–C) Pre-operation flexion and extension X-ray, the ROM of operating segments was 6.5°, and that of the upper adjacent segment was 1.7°. (D) Pre-operation T2WI MRI demonstrated L4/5 and L5/S1 disc herniation. E: Lateral X-ray at 32 months after surgery. (F–G) Flexion-extension X-ray at 32 months after surgery, the ROM of operating segments was 1.7°, and that of the upper adjacent segment was 4.7°. (H) T2WI MRI at 32 months after surgery.

Patient 1 was a male, aged 47 years, diagnosed with L4/5 and L5/ S1 lumbar disc herniation and spinal stenosis. Dynesys fixation was performed (Figure 1).

**Figure 2:**
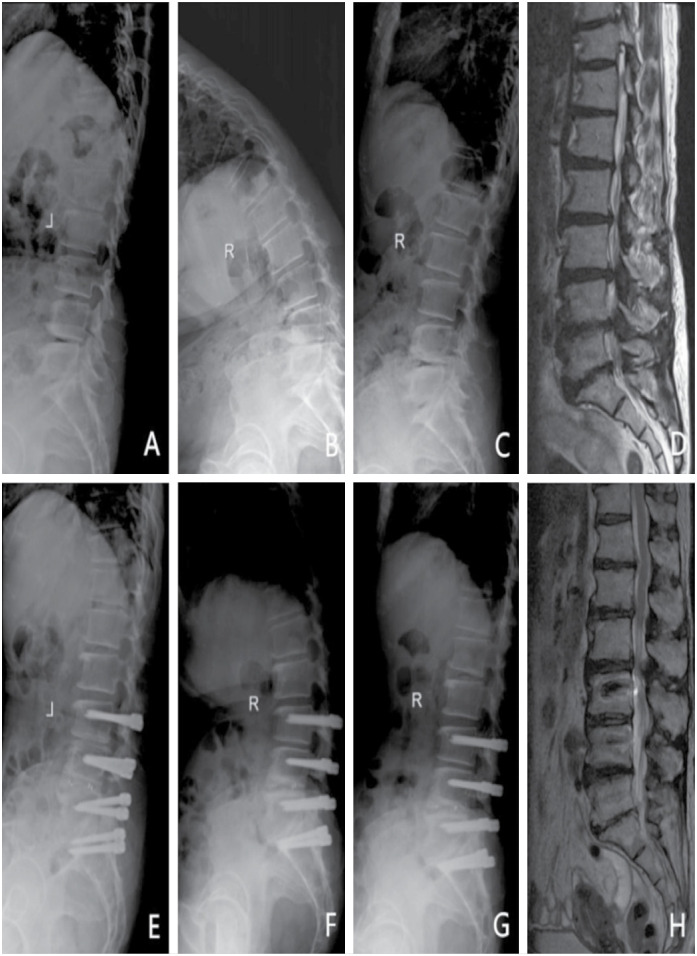
A 58-year-old female patient underwent surgery with a hybrid fixation system due to lumbar disc herniation and spinal stenosis in L3/4, L4/5, and L5/S1. (A) Pre-operation lateral X-ray. (B–C) Pre-operation flexion and extension X-ray, the ROM of operating segments was 40.0°, and that of the upper adjacent segment was 7.9°. (D) Pre-operation T2WI MRI demonstrated L3/4, L4/5, and L5/S1 disc herniation. (E) Lateral X-ray at 43 months after surgery. (F–G) Flexion-extension X-ray at 43 months after surgery, the ROM of operating segments was 2.6°, and that of the upper adjacent segment was 10.5°. (H) T2WI MRI at 43 months after surgery.

Patient 2 was a female, aged 58 years, diagnosed with L3/4, L4/5, and L5/S1 lumbar disc herniation and spinal stenosis. hybrid fixation was performed (Figure 2).

## DISCUSSION

### Symptomatic relief, and functional improvement of LDD patients

All patients revealed appreciable symptomatic relief and functional improvement during the follow-up period. Although there were much fewer Dynesys fixation and hybrid fixation surgeries than fusion surgeries, the clinical efficacy of Dynesys fixation and hybrid fixation for multi-level LDD has been proven by several studies. Hu et al. compared Dynesys fixation and rigid fusion after five years of follow-up and demonstrated that both groups experienced equally improvement in ODI and VAS.^11^ In a two-year study, similar results were found.^12^ Hu et al. also compared hybrid fixation and fusion surgery in their study and found that both groups reported comparable decline in ODI and VAS.^13^

### Influence on ROM and DH in the operating segments

In the present study, the ROM in the operating segments of both groups was preserved. However, the Hybrid group reported smaller ROM at the final follow-up. The height of the intervertebral space of the operating segments in the Dynesys group stayed constant from pre-operation to one-week post-operation while continually decreasing afterward, correlating to the ROM of the operating segments. The Hybrid group experienced a similar progression in the DH of the fusion segment and dynamic segment as the Dynesys group. However, there was no statistically significant change in the DH of the Hybrid group between the final follow-up and the baseline. At the same time, our findings demonstrated that the DH of dynamic segment of the Hybrid group had a significantly smaller change from one-week post-operation to final follow-up than the Dynesys group. It could be due to the more limited ROM in the Hybrid group.

Other studies have also reported similar results. Five years after the surgery, the intervertebral space height in the Dynesys group was lower than pre-operation.^11^ An analogous outcome was reported by other researchers.^14^ A total of 27 patients who received hybrid fixation were included in the study by Hu et al.^13^ They also utilized Dynesys devices and interbody cages. Their report claimed that the DH of the fusion segment increased at the last follow-up than pre-operation, while it appeared to decrease in the dynamic segment. However, our study did not find any DH difference between pre-operation and final follow-up in either the fusion or the dynamic segment. The continuous degeneration in dynamic segment may take time to show in X-ray. Therefore, we may get a result similar to Hu et al. had we extended the follow-up duration.

### The prevalence of ASD

It remains controversial whether Dynesys fixation and hybrid fixation can prevent ASD. Theoretically, the Dynesys system can reduce the stress on the adjacent disc above the operating segment by moderating the movement of the adjacent segment, thereby staving off ASD. Under the restriction of the Dynesys device, however, there is no doubt that the ROM of the upper adjacent segment will grow.^15^ In our study, the ROM of the upper adjacent segment in both groups increased than baseline, and there was no significant difference between the two groups. Simultaneously, the height of the upper adjacent intervertebral space also decreased due to the extra stress. ASD may develop over time as a result of persistently exceeding the physiological limits of the upper adjacent segment.^16^ Sven et al. reported a 28.2% incidence of ASD in their study after a 7.2-year follow-up.^17^ Hu reported that the incidence of ASD in the Hybrid fixation group was 18.5%.^13^

In this study, there was no difference in ASD between both groups. It is certain that ASD inevitably develops in patients after the two surgical procedures.

### The prevalence of other complications

SSI is not a rare complication for patients who received Dynesys fixation. A study reported wound infection rates of 2.22% after Dynesys fixation.^10^ The difference between the Hybrid group and the Dynesys group was not statistically significant in this study. Since hybrid fixation also used the Dynesys device, the surgeon should watch for SSI post-operation and react appropriately and promptly. Screw loosening is also a common complication of Dynesys fixation. In different retrospective studies, the incidence of screw loosening ranged from 18% to 19.8%.^18,19^ In our study, there was no significant difference in screw loosening between the two groups.

## CONCLUSION

We observed a significant improvement in VAS and ODI in each group. Both of them could preserve the ROM of stabilized segments, although Dynesys fixation allows a larger ROM, whereas hybrid fixation is better at maintaining the disc height of the dynamic level. The authors feel that both surgical procedures are effective treatments for middle-aged and physically active patients with multi-segmental LDD.
